# Correcting vaccine misinformation: A failure to replicate familiarity or fear-driven backfire effects

**DOI:** 10.1371/journal.pone.0281140

**Published:** 2023-04-12

**Authors:** Ullrich K. H. Ecker, Caitlin X. M. Sharkey, Briony Swire-Thompson

**Affiliations:** 1 School of Psychological Science, University of Western Australia, Perth, Australia; 2 Public Policy Institute, University of Western Australia, Perth, Australia; 3 Network Science Institute, Northeastern University, Boston, MA, United States of America; 4 Institute of Quantitative Social Science, Harvard University, Cambridge, MA, United States of America; University of Hull, UNITED KINGDOM

## Abstract

Individuals often continue to rely on misinformation in their reasoning and decision making even after it has been corrected. This is known as the continued influence effect, and one of its presumed drivers is misinformation familiarity. As continued influence can promote misguided or unsafe behaviours, it is important to find ways to minimize the effect by designing more effective corrections. It has been argued that correction effectiveness is reduced if the correction repeats the to-be-debunked misinformation, thereby boosting its familiarity. Some have even suggested that this familiarity boost may cause a correction to inadvertently *increase* subsequent misinformation reliance; a phenomenon termed the familiarity backfire effect. A study by Pluviano et al. (2017) found evidence for this phenomenon using vaccine-related stimuli. The authors found that repeating vaccine “myths” and contrasting them with corresponding facts backfired relative to a control condition, ironically increasing false vaccine beliefs. The present study sought to replicate and extend this study. We included four conditions from the original Pluviano et al. study: the myths vs. facts, a visual infographic, a fear appeal, and a control condition. The present study also added a “myths-only” condition, which simply repeated false claims and labelled them as false; theoretically, this condition should be most likely to produce familiarity backfire. Participants received vaccine-myth corrections and were tested immediately post-correction, and again after a seven-day delay. We found that the myths vs. facts condition reduced vaccine misconceptions. None of the conditions increased vaccine misconceptions relative to control at either timepoint, or relative to a pre-intervention baseline; thus, no backfire effects were observed. This failure to replicate adds to the mounting evidence against familiarity backfire effects and has implications for vaccination communications and the design of debunking interventions.

## Introduction

Misinformation—used here as an umbrella term for any information that is objectively false—has been identified as a serious issue for contemporary societies. This is not least because beliefs formed from invalid information may lead to behaviours that are potentially harmful or undesirable [1–3]. Vaccine misinformation is a prime example of this, as illustrated by the negative effect of false information about the mumps-measles-rubella (MMR) vaccine, or more recently the COVID-19 vaccines, on uptake rates [4, 5]. One of the insidious characteristics of misinformation is that it can continue to influence people’s reasoning and decision making even after it has been credibly corrected, a phenomenon known as the continued influence effect [6–8]. The persistent nature of misinformation has attracted much research seeking to examine the best methods to debunk “myths” in a way that most effectively reduces their subsequent impact (note we use the term “myth” to refer to a piece of common real-world misinformation) [9]. To this end, the present study sought to replicate a vaccine misinformation study by Pluviano et al. (2017) [10], who reported a failure of three debunking strategies.

It is generally acknowledged that the continued influence effect is at least partially based on failures of memory updating and retrieval processes [8]. One specific theoretical account—drawing on dual-process theories of memory [11]—posits that reliance on corrected misinformation occurs when a cue triggers retrieval of the misinformation based on its familiarity, but without recollection of the corresponding correction [12]. The familiarity of a myth has therefore been suggested as a driver of continued influence [13]. It is also well-known that repetition of information makes it more familiar and thereby more believable. This phenomenon is known as the illusory truth effect [14–16]. This effect occurs whether the information is true or false, and even if information conflicts with existing, factual knowledge [17, 18]. Concern over illusory truth effects has led to the assumption that repeating misinformation within a correction may render the correction less effective by boosting the familiarity of the misinformation being corrected. Some have even argued that corrections can backfire due to the boost to the misinformation’s familiarity, and ironically increase the very misconception they are designed to reduce, relative to either a pre-correction baseline or a no-correction control group [19, 20]. Demonstrations of such backfire effects have led to recommendations to avoid misinformation repetition when debunking misinformation [19, 21, 22].

However, as reviewed in detail elsewhere [8, 23], the evidence for such familiarity backfire effects is actually quite weak: (1) The most cited study reporting familiarity backfire [Skurnik et al., 2007 [unpublished]; summarized in 20] is not accessible as a preprint. (2) Many studies claiming to have found familiarity backfire in fact only demonstrate a to-be-expected belief regression post-correction (i.e., a correction initially reduces belief and this corrective effect slowly wears off over time, with belief returning back to baseline; [24, 25, but see 26 for a failed replication]). (3) There is ample evidence that familiarity backfire effects do not emerge even under conditions designed to be maximally conducive [13, 27–29]. For example, Swire et al. (2017) presented participants with real-world myths, and corrected them using either brief or detailed explanations, which resulted in each false claim being presented three times during the experiment (thus boosting claim familiarity). Swire et al. tested young and older adults and varied the study-test delay from minutes to three weeks—the rationale being that (i) older adults should be more susceptible to familiarity effects because their ability to recollect details of the correction should be impaired, whereas familiarity-based memory is relatively unaffected by age [30], and that (ii) substantial delays should promote familiarity effects because recollection is affected more strongly by delays than familiarity [11]. However, corrections *reduced* belief in false claims in all conditions—even when the corrections were scant on detail, in older adults, and after a three-week delay. Some have argued that familiarity backfire effects are mainly a concern with novel misinformation, because a correction may then introduce a person to a false claim they have never encountered before (thus providing a maximal familiarity boost, so to speak) [22]. However, evidence for this is also mixed at best [31–33].

One of the best pieces of evidence for familiarity backfire effects is a study by Pluviano et al. (2017) [10]. Pluviano and colleagues investigated how corrections of childhood vaccine myths impacted (i) concerns about vaccine side effects, (ii) belief in the debunked link between the MMR vaccine and autism, as well as (iii) vaccination intention (vaccine hesitancy). The study randomly assigned participants to one of four conditions: a common vaccine “myths-versus-facts” condition; a visual-correction condition utilising an infographic comparing disease and vaccine risks; a fear-appeal condition using images of sick (unvaccinated) children; or a control condition presenting unrelated fact sheets about healthcare. Participants’ vaccine-myth beliefs and vaccination intentions were measured immediately after receiving a correction intervention (Time 1; T1), and again after a one-week delay (Time 2; T2), using the same questionnaire (note that a baseline measure using a more generic questionnaire was obtained before the intervention [Time 0; T0]; however, this measure was not included in any of Pluviano et al.’s analyses). None of the interventions substantially reduced misconceptions concerning vaccines relative to control. Instead, the myths-vs.-facts condition appeared to increase participants’ belief in vaccine side effects and the vaccine-autism link, as well as reducing their intention to vaccinate, at T2. In a subsequent study, Pluviano et al. (2019) replicated this pattern of results in a parent population [34], where participants in a myths-vs.-facts condition held stronger misconceptions regarding vaccine side effects and the vaccine-autism link compared to control. This can be interpreted as evidence for familiarity backfire because the myths-vs.-facts format explicitly repeated the misinformation, boosting its familiarity. However, it should also be noted that the fear appeal in Pluviano et al. (2017) also backfired, although this may have been for different reasons, most likely a misattribution of emotional arousal (see below).

The selection of conditions in Pluviano et al. (2017) was well-considered from both applied and theoretical perspectives. First, the myths-vs.-facts format is a very common format to address misconceptions in the real world, for example via posters or pamphlets. It is also theoretically interesting: On the one hand, offering an explanation about why a myth is false is a key ingredient of an effective correction, particularly when also providing a factual alternative [6, 9, 29, 35]. On the other hand, the format has the potential to backfire because it involves repetition of the misinformation.

Second, visual interventions such as infographics are also commonly used in attempts to correct misinformation. They promise persuasiveness through attention capture and engagement and effective communication of complex concepts (e.g., weight-of-evidence messages and scientific belief representations) [36, 37] that leaves little room for misinterpretation and counterarguments [38–40].

Finally, fear appeals are sometimes used in misinformation interventions where there is a relevant and significant threat. In such cases, fear appeals have been shown to be effective as long as there is high self-efficacy (i.e., the recipient has a sense that they can actively do something to avert the threat, such as quit smoking) [41–43]. In addition, emotive images used in fear appeals may increase their overall persuasiveness [44]. However, in line with Pluviano et al. (2017), Nyhan et al. (2014) [45] found that providing images of sick children depicting the symptoms of disease in a pro-vaccination campaign may be counterproductive, as misattribution of emotional arousal can potentially increase vaccine concerns [46].

### The present study

Although the focus of our theoretical interest was on the familiarity backfire effect (and thus the comparison of myths-vs.-facts and control conditions), it was decided to include all of Pluviano et al.’s (2017) conditions. The present study thus replicated the Pluviano et al. (2017) study design, with several methodological enhancements. First, we additionally included a “myths-only” condition, which used the same materials as the myths-vs.-facts condition, but only presented the myths—labelled as such—without the facts. This format is an example of a weak, terse retraction that provides minimal correctional detail to recall later, and as such should be particularly likely to produce familiarity backfire [6, 12]. Second, we included questions at baseline (T0) that were also given immediately post-correction (T1) and after a one-week delay (T2); this allowed for a within-subjects pre-post intervention comparison in addition to the between-subjects comparison between correction and no-correction conditions, to better establish the potential presence of a familiarity backfire effect. Third, we used multi-item measures instead of single-item measures to assess the dependent variables, because it is known that single-item measures often lack reliability, and their use has been causally related to observations of backfire [23, 33].

A final change was motivated by the possibility that the backfire effect reported by Pluviano et al. (2017) was driven by worldview rather than familiarity. Such worldview backfire effects are occasionally observed when a correction challenges a misconception that a person is motivated to protect for ideological reasons [47, 48]. These effects have also been difficult to replicate [49–52], but it is conceivable that worldview was an important factor in Pluviano et al.’s (2017) study. This is because worldview backfire effects have previously been found with vaccine stimuli [45, 53] (but see [54]) and because Pluviano et al.’s sample was drawn from Italy and the UK, where vaccine hesitancy levels were relatively high at the time the study was conducted [55, 56]. Thus, we added an empirically-tested scale to assess vaccination attitudes, alongside a measure of identity centrality that assessed the importance of the vaccine attitude to the individual, as it has been suggested that only attitudes that are a central part of an individual’s identity significantly impact reasoning [23, 47]. Thus, a compound measure of vaccination attitudes and identity centrality was used as a covariate in the analyses, and also to allow for focused analysis of a subsample with relatively high vaccine concern, which may show worldview backfire effects.

Although Pluviano et al. (2017) found evidence for familiarity backfire effects, considering the overall body of research reviewed earlier, no backfire effect was expected. Therefore it was hypothesized that in the myths-only and myths-vs.-facts conditions, participants’ beliefs in vaccine side effects, the vaccine-autism link, and vaccination hesitancy would be lower than control at both T1 and T2. It was also expected that there would be an initial decrease from T0 to T1 immediately post-correction, which, however, would not be fully sustained over time [13, 23]; as such, it was expected that there would be an increase from T1 to T2. Regarding the other conditions, there was no reason to believe the visual correction would backfire, and thus it was expected that this condition would also be effective at reducing misconceptions. Finally, we had no strong expectations regarding the fear-appeal condition, given the inconsistent evidence from previous research, but again hypothesized that there would be no backfire. In sum, no experimental condition was predicted at T1 or T2 to exceed baseline levels at T0 or the control condition at T1 and T2, respectively.

## Method

The core study design comprised the between-subjects factor condition with five levels (control; myths-only; myths vs. facts; visual correction; fear appeal) and the within-subjects factor time with two levels (immediate post-test, T1; delayed test, T2). Three dependent variables were measured (concern with vaccine side-effects; belief in the autism-vaccine link; vaccination hesitancy) with seven items each. A subset of three items (one per dependent variable) was additionally administered at baseline (T0) to allow for a pre-post comparison. Vaccine attitudes and their identity centrality were measured at T0.

### Participants

An a-priori power analysis using G*Power 3 [57] suggested a minimum sample size of 64 per condition to detect a difference of effect size *f* = 0.25 (with α = 0.05; 1 –β = 0.80) in between-subjects *F*-tests between the myths-vs.-facts and control conditions—the main comparisons of interest (note that the effect size was determined somewhat arbitrarily, but set to be smaller than the relatively large effect sizes reported by Pluviano et al. We also acknowledge that the *F*-tests referred to here are slightly different from the contrasts performed in the Results section (which were planned contrasts that take the full ANOVA model with all conditions into account and were subject to Holm-Bonferroni correction, which reduced achieved power). To ensure ample power and to account for exclusions (see below), it was decided to aim for 75 participants per condition, or a total sample size of 375. To additionally account for an expected drop-out rate of 15% between T1 and T2, a convenience sample of 440 UK-based participants was recruited using Prolific. Of these, 383 participants completed both parts of the study. Based on a-priori exclusion criteria (see below), data of three participants were excluded, leaving a final sample of *N* = 380 (95 males, 283 females, 2 non-binary participants; mean age was *M* = 36.45 years [*SD* = 11.66], age range was 18–76). This sample size was large compared to Pluviano et al.’ studies (2017, *N* = 120; 2019, *N* = 60). At the time of the study, 110 participants (29%) had a child under the age of six; this information was obtained to assess results in the parent population specifically, allowing for a comparison with Pluviano et al. (2019). Upon completion, participants received a compensation of £1 for Part 1 and £0.90 for Part 2.

### Materials

#### Stimuli

Stimuli were taken directly from Pluviano et al. (2017) and are provided in the S1 File, available at https://osf.io/dwyma/.

*Myths vs*. *facts*. Ten common vaccine misconceptions (“myths”) were juxtaposed against 10 corresponding facts taken from World Health Organization educational materials (see S1 Table in S1 File). An example is “MYTH: Natural immunity is better than vaccine-acquired immunity. Indeed, catching a disease and then getting sick results in a stronger immunity to the disease than a vaccination.” vs. “FACT: Vaccines interact with the immune system to produce a response similar to that produced by the natural infection, but they protect against its potential severe complications.” Each myth/fact pair was presented on a separate page, with the fact appearing directly beneath the myth.

*Myths only*. In this condition, only the 10 vaccine myths were presented, without the corresponding facts. Each was labelled explicitly as a myth and presented on an individual page. This condition was not part of the original Pluviano et al. (2017) study.

*Visual correction*. In this condition, corrections visually compared the potential risk of symptoms if infected with a vaccine-preventable disease against the risk of vaccine side-effects. Individual diagrams for measles, mumps, and rubella were used, with each diagram showing 100 coloured stick figures to represent the degree of complications experienced—green (no/mild symptoms); yellow (moderate complications); and red (serious complications). This was supplemented by a short written explanation outlining the probability of experiencing these specific symptoms.

*Fear appeal*. In this condition, participants were shown three photographs depicting unvaccinated children with symptoms of mumps, measles, and rubella. Images were accompanied by a personalized written warning stating that “you will see some of the consequences you may face by choosing to not vaccinate your child”. It also featured a series of dot points providing details about disease-specific infection risk and symptoms (e.g., “The measles virus can be spread very easily”; “Measles also can cause pneumonia, brain damage, seizures or death”).

*Control*. Two fact sheets unrelated to vaccination safety were used in the control condition. One sheet contained 20 tips on how to prevent medical errors, while the other outlined five steps to safer healthcare. Participants viewed both fact sheets.

#### Measures

*Pre-manipulation survey*. The pre-manipulation survey administered at T0 contained three items assessing participants’ baseline side-effect concerns, belief in a vaccine-autism link, and vaccine hesitancy. These items were: “I am concerned about serious adverse effects of vaccines”; “Some vaccines cause autism in healthy children”; and “Getting vaccines is a good way to protect my future child(ren) from disease”. Participants responded on Likert scales ranging from 0–5 (*strongly disagree–strongly agree*). Pluviano et al. (2017) also administered a pre-manipulation survey including these three items (plus five other items assessing general vaccine attitudes, which were assessed in the present study at T0 with the dedicated 12-item Vaccination Attitude Examination scale described below). Although Pluviano et al. did not report any results from the pre-manipulation survey, we included it because (i) it may have provided some framing or priming that potentially influenced results in Pluviano et al.’s study, and (ii) because in the present study, the baseline items were also included in the post-manipulation survey, allowing for a direct pre-post comparison between T0 and both T1 and T2.

*Post-manipulation survey*. The 21-item post-manipulation survey was more specific to the intervention materials; it used seven items each to assess (i) belief in side effects (two items reverse-coded), (ii) the vaccine-autism link (three items reverse-coded), and (iii) vaccine hesitancy (four items reverse-coded), respectively. Three items were taken directly from the Pluviano et al. (2017) materials (one per measure: “How likely is it that children who get the measles, mumps, and rubella [MMR] vaccine will suffer serious side effects?”; “Some vaccines cause autism in healthy children.”; and “How likely is it that you would give your future child(ren) the MMR vaccine?”). These items were presented first, to allow for a direct replication of the Pluviano et al. analyses; these items were supplemented by new additional items (six per measure) to increase reliability. Responses were recorded on Likert scales ranging from 0–5 (*strongly disagree–strongly agree* or *very unlikely–very likely*). The post-manipulation survey was administered twice—once immediately post-intervention at T1, and again after a one-week delay at T2.

*Vaccination attitude examination (VAX scale)*. The VAX scale [58] consists of twelve items assessing general vaccine attitudes, including mistrust of vaccine benefits, worries about unforeseen future effects, concerns about commercial profiteering, and a preference for natural immunity. An example item is “I feel safe after being vaccinated”. Responses were recorded on Likert scales ranging from 0–5 (*strongly disagree–strongly agree*); three items were reverse-coded. The VAX scale has high internal consistency (α = .92) and good convergent and construct validity [59].

*Identity-centrality survey*. To assess the importance of vaccine beliefs and attitudes to participants’ identity, two items were administered. These items were “My views about vaccinations are central to my identity” and “Vaccinations are an important topic to me”. Responses were measured on Likert scales ranging from 0–5 (*strongly disagree–strongly agree*). A participant’s score on the identity centrality scale was multiplied by their VAX *z*-score; this compound measure was then *z*-transformed to create a “VAX-ID” vaccination-attitude score that was used as a covariate in the analyses.

### Procedure

The experiment was approved by the Human Research Ethics Office of the University of Western Australia (RA/4/20/6423). It was conducted online in May/June 2021, and administered using Qualtrics survey software (Qualtrics, Provo, UT). Participants initially received an information sheet and provided informed consent by ticking a box in the online survey before the study commenced. The information provided explained that the study was unrelated to COVID-19. Participants then (T0) answered the demographic questions (age, gender, and whether they had any children under the age of six). This was followed by the VAX scale, which used a fixed question order, as well as the identity-centrality scale and pre-manipulation survey, both of which used a randomised question order. Participants were then randomly assigned to one of the five conditions (control; myths-only; myths vs. facts; visual correction; fear appeal). After being presented with the respective intervention materials, all participants completed the post-manipulation survey (T1). In the post-manipulation survey, the three items taken from Pluviano et al. (2017) were always presented first, followed by the 18 new items in a quasi-random order (note that to minimize the number of response-scale switches, the original item using an agree/disagree response scale was presented first, followed by the two original items using a likely/unlikely response scale; this was followed by the two new questions using a likely/unlikely response scale [in random order] and finally the 16 new items that used an agree/disagree scale [also in random order]). After a week (T2), participants were invited back to complete Phase 2 of the study, where they were presented with the post-manipulation survey again. Phase 2 was open for ~ 48 hours. All stimuli and survey questions were presented for set minimum times (approx. 150 ms per word) to ensure that participants spent an adequate amount of time engaging with the written materials and questions. At the conclusion of the study, participants were asked whether their data should be used or discarded due to lack of effort, and were then fully debriefed. The debriefing explained to participants that they may have been exposed to vaccine misinformation and how this may affect them. They were also given the ten facts from the myths-vs.-facts condition and links to relevant World Health Organization and National Health Service web pages (see S1 File). The experiment took approximately 15 minutes to complete (10 minutes for Phase 1; 5 minutes for Phase 2).

## Results

Data were excluded from analysis based on a-priori criteria. Specifically, we first screened for participants who indicated their data should be discarded due to lack of effort (*n* = 0) and those who showed uniform responding (*SD* < 0.5 across all rating-scale items; *n* = 0). Scores of reverse-coded items were then reversed, and data were screened for inconsistent responding; this was done by (i) computing separate means for reverse-coded and regular items for the VAX scale and each of the three dependent measures at each of the two time-points, (ii) computing a grand mean from the seven absolute differences between those means, and (iii) applying the outlier-labelling rule with a 2.2 multiplier [60] to identify outliers on that score (*n* = 3). We also assessed reliability and found that the VAX scale and each dependent-variable scale demonstrated very good internal consistency (all Cronbach’s α ≥ .87).

### Primary analyses: Side effects, vaccine-autism link, vaccination hesitancy

We first present the primary analyses of the three dependent variables measured using our multi-item scales and including our “VAX-ID” covariate. Supplementary analyses taking into account vaccination attitudes and parental status, analyses focused only on Pluviano et al.’s (2017) original items (i.e., a direct replication), and pre-post analyses are presented in a later section.

Three two-factorial within-between analyses of covariance (ANCOVAs) were conducted in order to examine whether beliefs in vaccine side effects, the vaccine-autism link, and vaccination hesitancy differed across time points and experimental conditions. The within-subjects factor time had two levels, T1 and T2; the between-subjects factor condition had five levels, reflecting the control, myths-only, myths-vs.-facts, visual-correction, and fear-appeal conditions. In order to take into account both participants’ general vaccination attitudes and the identity centrality of those attitudes, the VAX-ID covariate was included in a full-factorial model (note that ANOVAs without the covariate yielded equivalent results unless noted otherwise).

**Fig 1:**
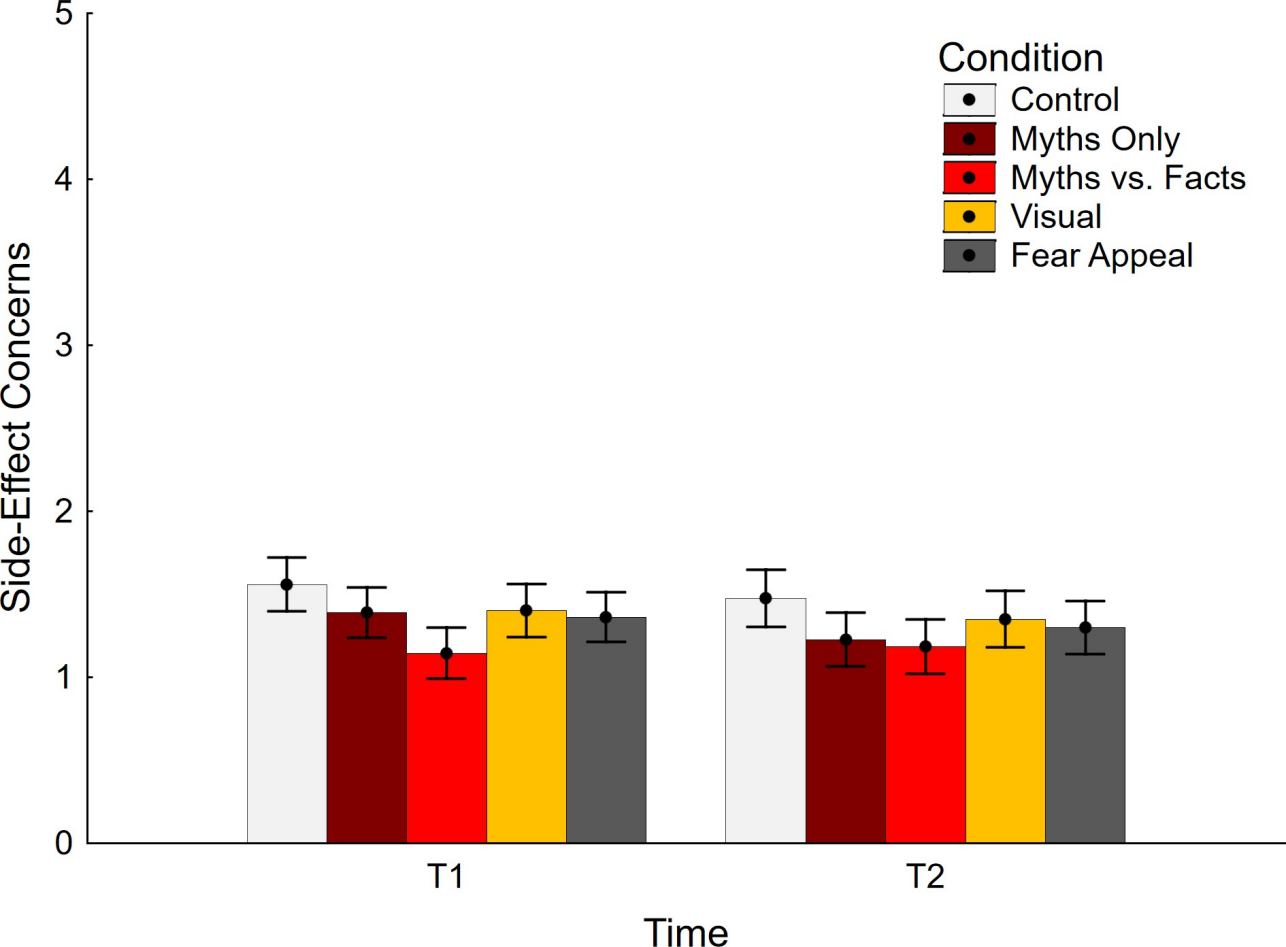
Concerns about side effects across conditions. Error bars show 95% confidence intervals.

Side-effect concern data are shown in Fig 1. The ANCOVA yielded a significant main effect of time, *F*(1, 370) = 5.45, *p* = .020, η_p_^2^ = .015, indicating slightly lower scores at T2 relative to T1. The main effect of condition was also significant, *F*(4, 370) = 2.73, *p* = .029, η_p_^2^ = .029, indicating a difference between test conditions (note that the effect was nonsignificant in an ANOVA, *F*(4, 375) = 2.34, *p* = .055, η_p_^2^ = .024). The interaction was not significant, *F*(4, 370) = 1.44, *p* = .218, η_p_^2^ = .015. As expected, the covariate had a significant impact, *F*(1, 370) = 336.61, *p* < .001, η_p_^2^ = .476, but was not involved in any interactions, all *F*(1/4, 370) ≤ 3.18, *p* ≥ .075, η_p_^2^ ≤ .009.

Planned contrasts were conducted to compare each experimental condition against control at each delay; these are presented in Table 1 (top section). The analyses revealed that the myths-vs.-facts condition was associated with reduced concern about vaccine side effects at Time 1 relative to control. In other words, this condition was effective at reducing side-effect concerns. No conditions were associated with elevated concerns relative to control at any time. This indicated that there was no backfire effect in any of the conditions.

**Table 1:** Contrast analyses.

Contrast (vs. control)	*F*(1, 370)	η_p_^2^	*p*
Side Effects T1			
Myths Only	2.29	.006	.131
Myths vs. Facts	13.43	.035	< .001*
Visual	1.86	.005	.173
Fear Appeal	3.10	.008	.079
Side Effects T2		
Myths Only	4.24	.011	.040
Myths vs. Facts	5.71	.015	.017
Visual	1.02	.003	.313
Fear Appeal	2.11	.006	.147
Vaccine-Autism Link T1			
Myths Only	1.80	.005	.180
Myths vs. Facts	6.90	.018	.009*
Visual	0.13	< .001	.719
Fear Appeal	0.02	< .001	.884
Vaccine-Autism Link T2			
Myths Only	5.85	.016	.016
Myths vs. Facts	4.99	.013	.026
Visual	0.74	.002	.392
Fear Appeal	1.07	.003	.303
Vaccination Hesitancy T1			
Myths Only	0.03	< .001	.860
Myths vs. Facts	0.46	.001	.497
Visual	0.44	.001	.509
Fear Appeal	1.45	.004	.230
Vaccination Hesitancy T2			
Myths Only	1.75	.005	.186
Myths vs. Facts	0.99	.003	.320
Visual	0.59	.002	.443
Fear Appeal	0.10	< .001	.757

* indicates statistical significance following Holm-Bonferroni correction. Note that correction was applied to sets of contrasts defined by the combination of dependent variable and timepoint (i.e., family size 4), as per the a-priori analysis plan; however, one could also argue that correction should instead control only for the dual tests across timepoints (i.e., family size 2), as only those test the same hypothesis (e.g., “myths-only differs from control”; see [61]). This would result in some non-significant contrasts becoming significant.

**Fig 2:**
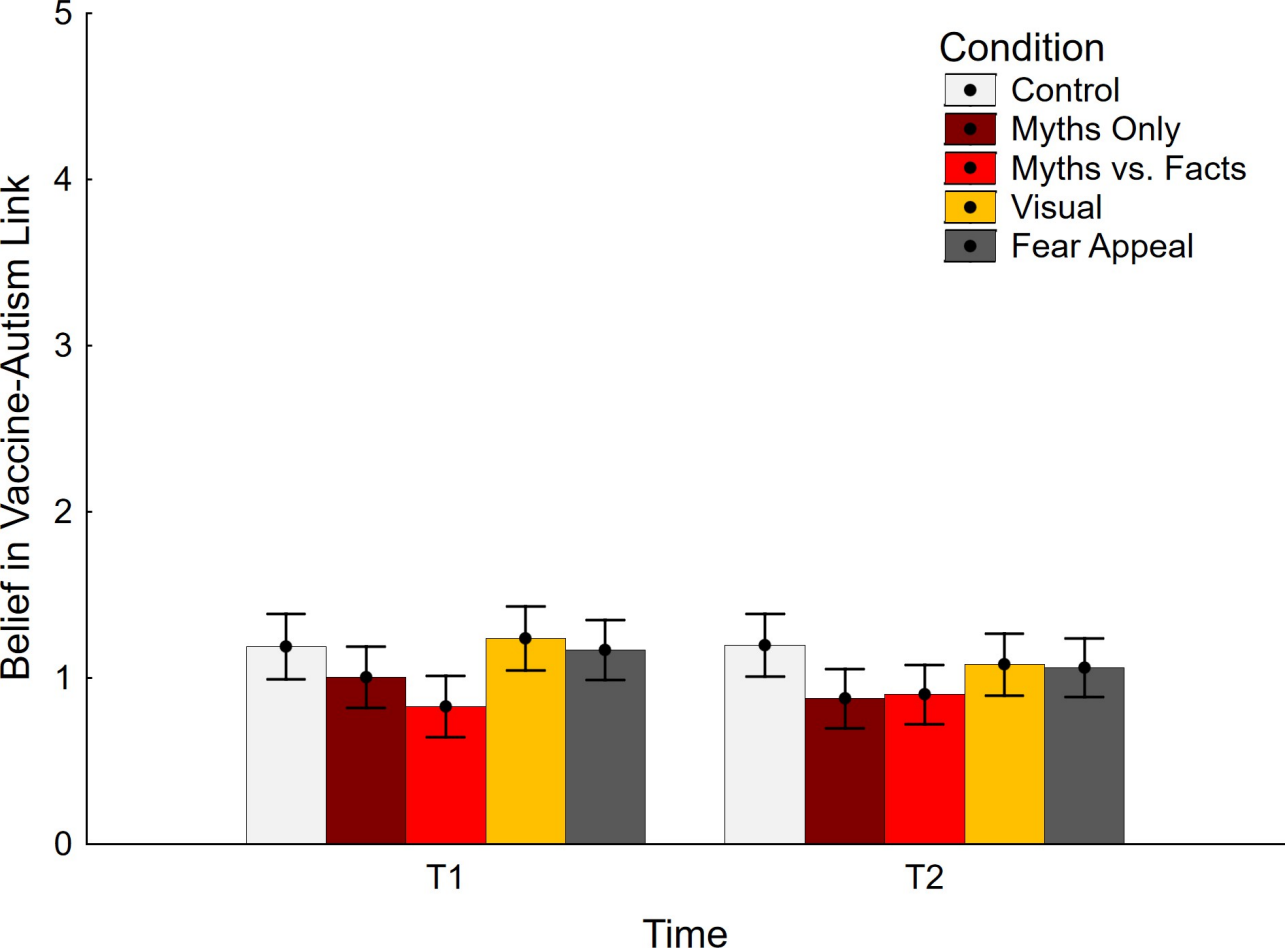
Belief in vaccine-autism link across conditions. Error bars show 95% confidence intervals.

Data regarding belief in the vaccine-autism link are shown in Fig 2. The ANCOVA yielded a main effect of time, *F*(1, 370) = 7.33, *p* = .007, η_p_^2^ = .019, indicating lower scores at T2 relative to T1. The main effect of condition was significant as well, *F*(4, 370) = 2.52, *p* = .041, η_p_^2^ = .027 (note that the effect was nonsignificant in an ANOVA, *F*(4, 375) = 2.27, *p* = .061, η_p_^2^ = .024). There was also a significant interaction of condition and time, *F*(4, 370) = 3.56, *p* = .007, η_p_^2^ = .037. The covariate had a significant impact, *F*(1, 370) = 264.72, *p* < .001, η_p_^2^ = .476, but was not involved in any interactions, all *F*(1/4, 370) ≤ 1.60, *p* ≥ .207, η_p_^2^ ≤ .006.

Planned contrast analyses showed that the myths-vs.-facts condition was associated with significantly lower belief in the vaccine-autism link at T1 (Table 1, middle section). No conditions were associated with a statistically significant belief increase relative to control at any time, indicating that there was no backfire effect present in any of the conditions.

**Fig 3:**
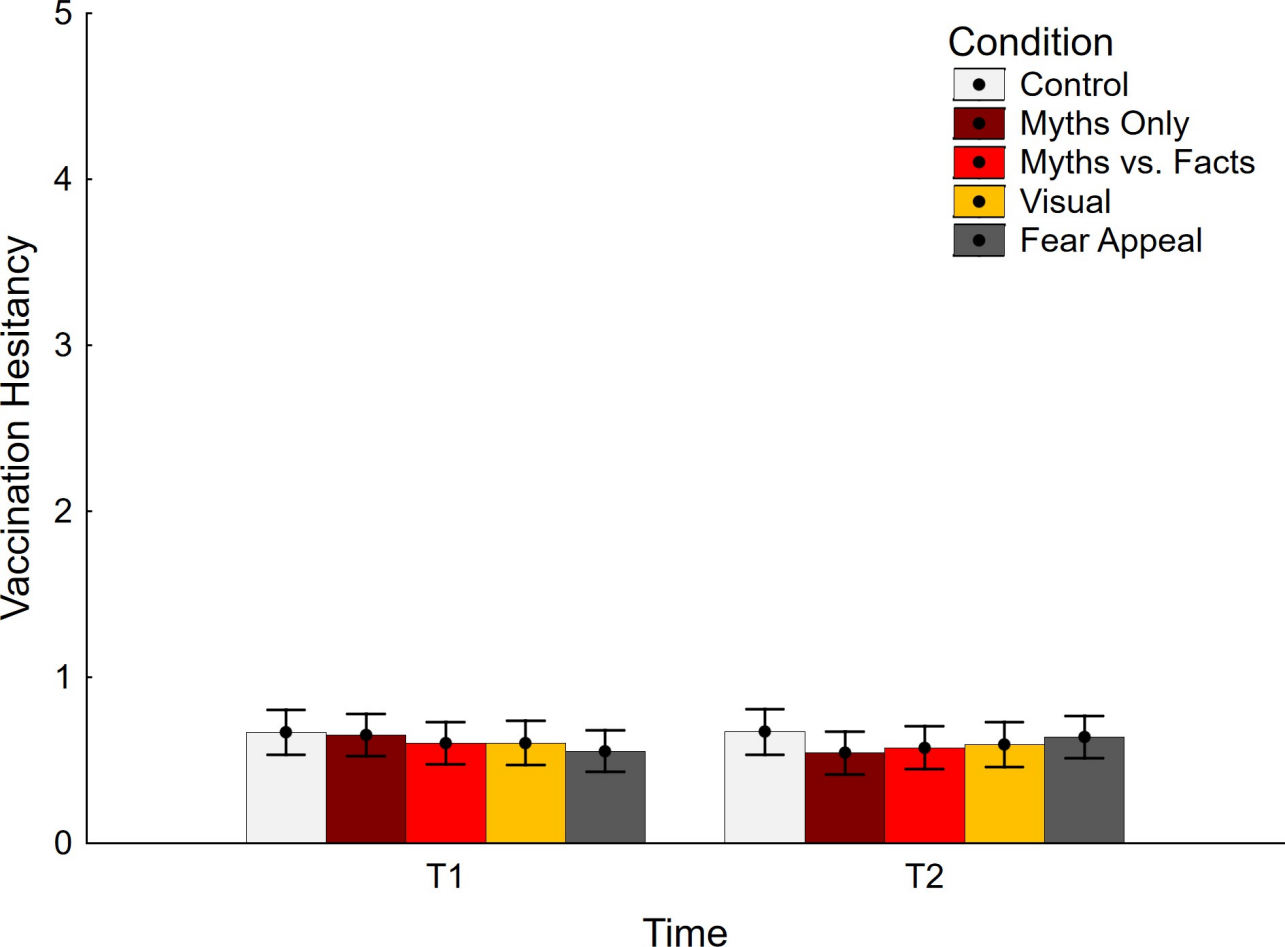
Vaccination hesitancy across conditions. Error bars show 95% confidence intervals.

Vaccination hesitancy results are presented in Fig 3. The ANCOVA returned non-significant main effects of time and condition, *F* < 1, but a significant interaction effect, *F*(4, 370) = 3.51, *p* = .008, η_p_^2^ = .037. However, no significant differences were found between control and the other experimental conditions, suggesting that no condition increased or decreased vaccine hesitancy, relative to control (refer to Table 1; bottom section). Again, this indicates that there was no backfire effect present in any condition.

### Supplementary analyses

#### Analyses considering vaccination attitudes

Given that the sample used in Pluviano et al. (2017) was drawn from a potentially more vaccine-hesitant population, supplementary analyses were performed on the top and bottom tertiles of the sample based on VAX-ID scores (*n* = 255). This involved repeated measures ANOVAs with the within-subjects factor time (T1, T2) and the between-subjects factors condition (control, myths only, myths vs. facts, visual, fear appeal) and VAX-ID group (top, bottom). As per a-priori analysis plan, this was followed by specific contrasts between control and experimental conditions in the top tertile regardless of ANOVA outcome, to ensure no potential backfire effect was missed. To foreshadow, no backfire effects emerged on any variable (note that exploratory analyses using more extreme groups [e.g., deciles] also found no evidence for backfire).

**Fig 4:**
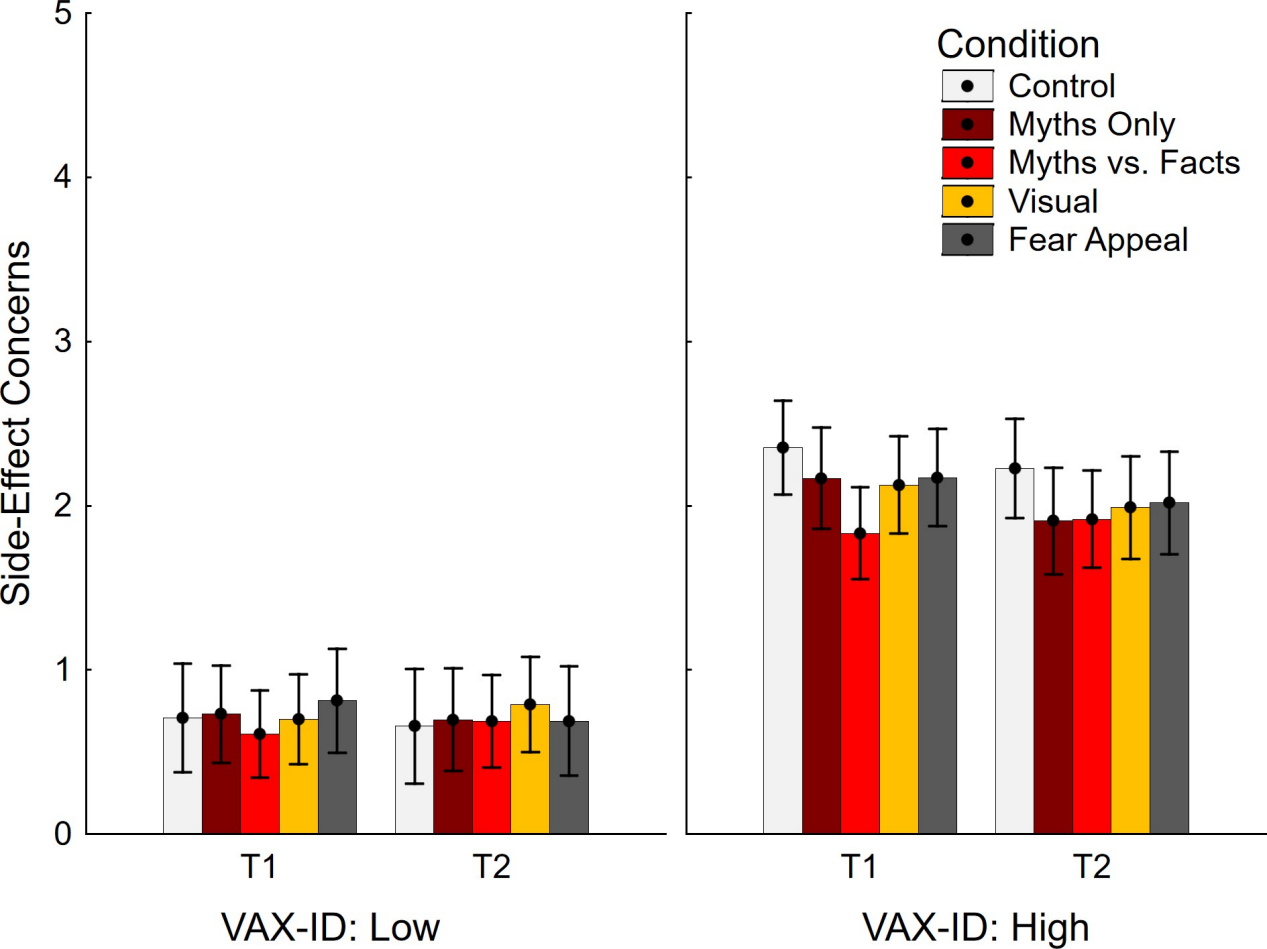
Concerns about side effects across conditions and VAX-ID groups. VAX-ID: product of vaccine-attitude and identity-centrality scores; T1: time 1; T2: time 2. Error bars show 95% confidence intervals.

Concerns about side effects are shown in Fig 4. There was only a significant main effect of VAX-ID group, in the expected direction, *F*(1, 245) = 221.28, *p* < .001, η_p_^2^ = .475. The main effects of time, *F*(1, 245) = 3.57, *p* = .060, η_p_^2^ = .014, and condition, *F* < 1, were non-significant, and there were no significant interactions, all *F*(1/4, 245) ≤ 2.71, *p* ≥ .101, η_p_^2^ ≤ .028. The planned contrast analysis focusing on the top tertile of vaccine-hesitant participants (see Table 2, top section) returned just one significant effect, suggesting reduced side-effect concerns in the myths-vs.-facts condition relative to control at T1.

**Table 2:** Contrast analyses in vaccine-hesitant group.

Contrast (vs. control)	*F*(1, 245)	η_p_^2^	*p*
Side Effects T1			
Myths Only	0.77	.003	.382
Myths vs. Facts	6.65	.026	.010*
Visual	1.20	.005	.274
Fear Appeal	0.77	.003	.381
Side Effects T2		
Myths Only	2.03	.008	.156
Myths vs. Facts	2.08	.008	.150
Visual	1.18	.005	.279
Fear Appeal	0.91	.004	.341
Vaccine-Autism Link T1			
Myths Only	3.41	.014	.066
Myths vs. Facts	2.94	.012	.088
Visual	0.29	.001	.594
Fear Appeal	< 0.01	< .001	.959
Vaccine-Autism Link T2			
Myths Only	2.20	.009	.139
Myths vs. Facts	0.78	.003	.377
Visual	0.01	< .001	.943
Fear Appeal	0.28	.001	.597
Vaccination Hesitancy T1			
Myths Only	0.01	< .001	.910
Myths vs. Facts	0.28	.001	.599
Visual	0.16	.001	.691
Fear Appeal	0.52	.002	.473
Vaccination Hesitancy T2			
Myths Only	0.34	.001	.561
Myths vs. Facts	0.80	.003	.371
Visual	0.39	.002	.533
Fear Appeal	< 0.01	< .001	.949

* indicates statistical significance following Holm-Bonferroni correction.

**Fig 5:**
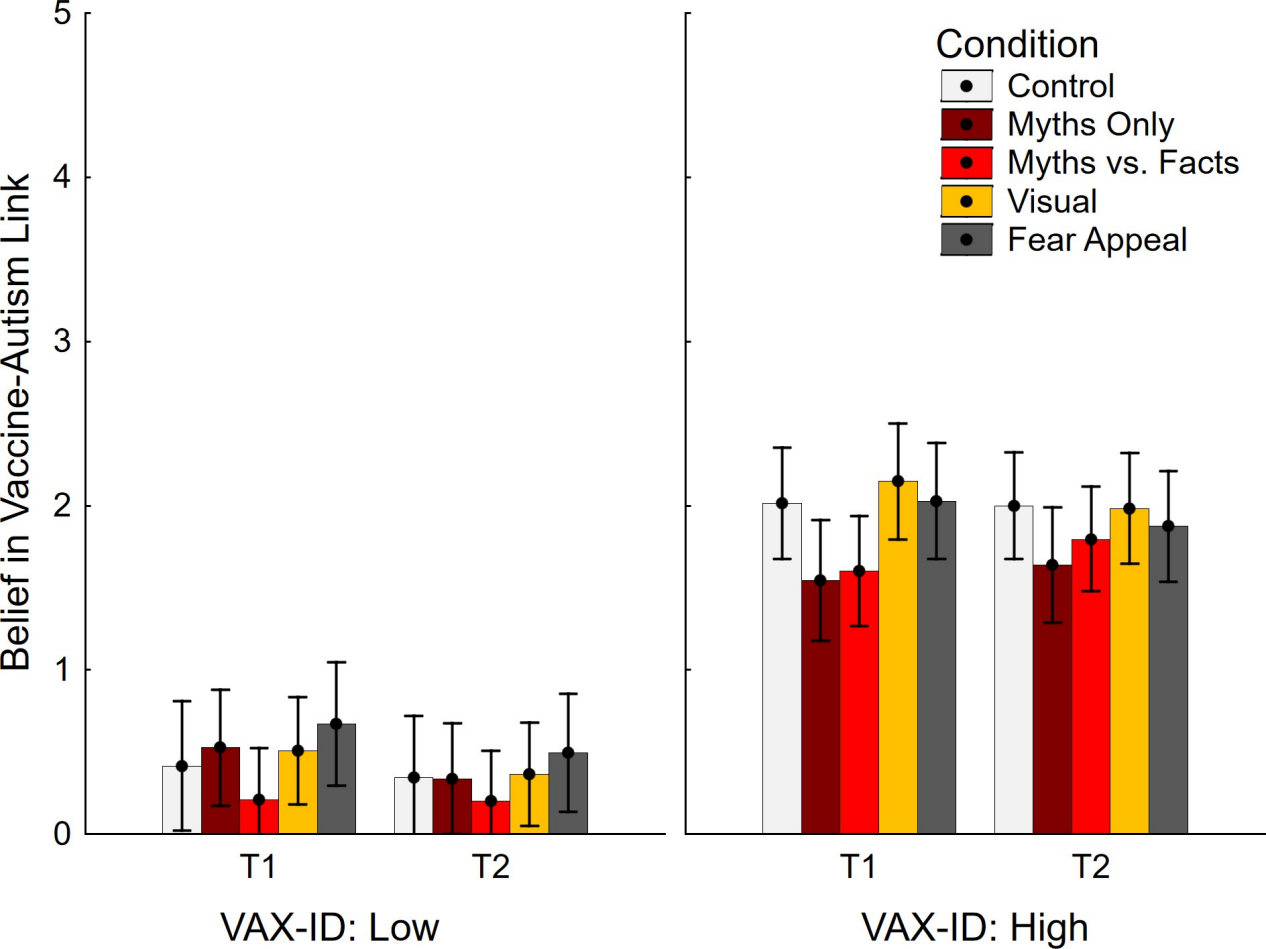
Belief in vaccine-autism link across conditions and VAX-ID groups. VAX-ID: product of vaccine-attitude and identity-centrality scores; T1: time 1; T2: time 2. Error bars show 95% confidence intervals.

Data regarding belief in the vaccine-autism link is shown in Fig 5. The ANOVA returned significant main effects of time, *F*(1, 245) = 5.17, *p* = .024, η_p_^2^ = .021, indicating slightly lower scores at T2 than T1, and VAX-ID group *F*(1, 245) = 185.37, *p* < .001, η_p_^2^ = .431. The main effect of condition was non-significant, *F*(4, 245) = 1.52, *p* = .197, η_p_^2^ = .024, but there was a time by condition interaction, *F*(4, 245) = 3.12, *p* = .016, η_p_^2^ = .048. No other interactions were significant, all *F*(1/4, 245) ≤ 3.70, *p* ≥ .056, η_p_^2^ ≤ .024. No contrasts were significant (see Table 2, middle section), indicating no significant impact of any interventions in the top tertile of vaccine-hesitant participants.

**Fig 6:**
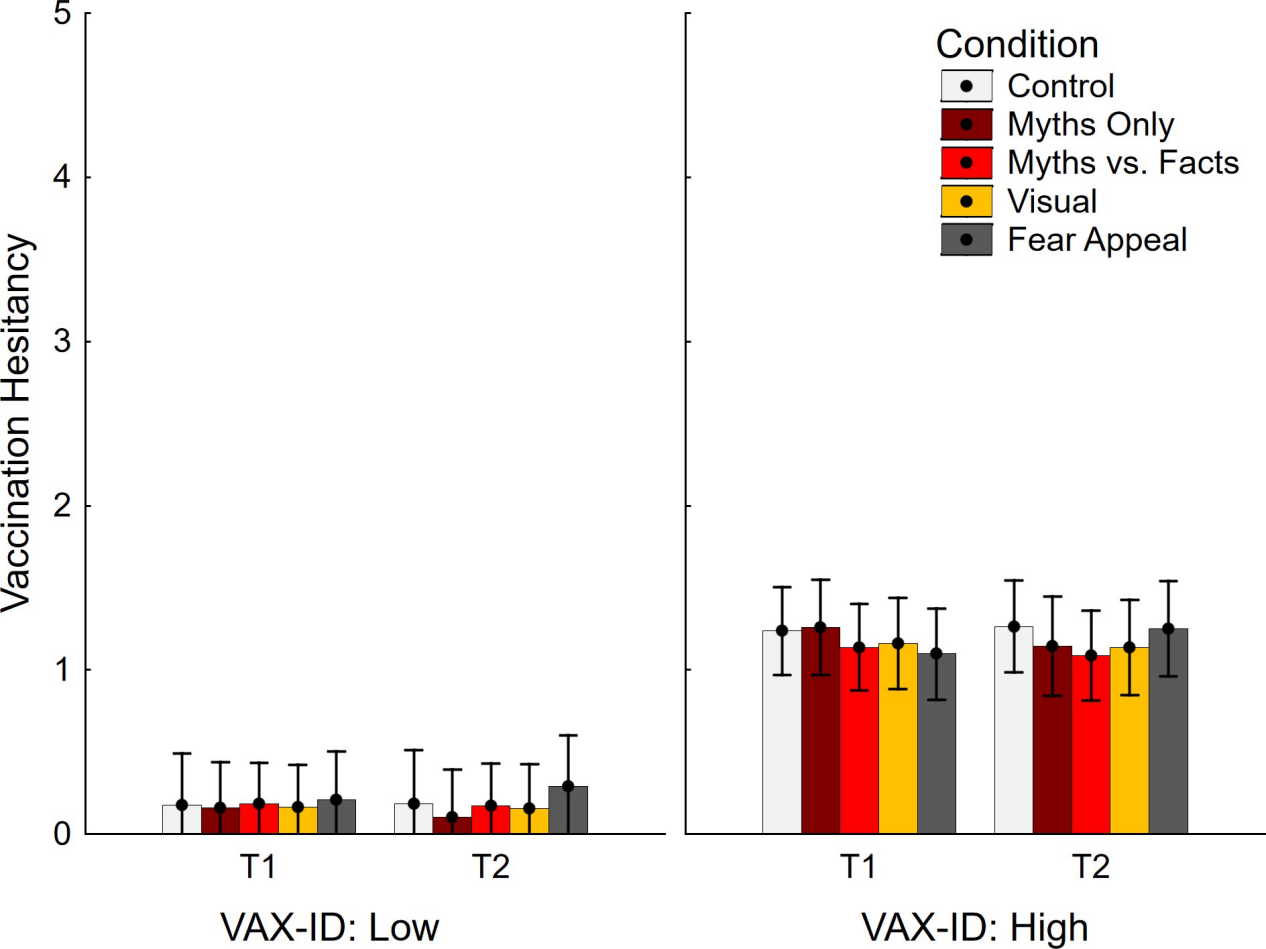
Vaccination hesitancy across conditions and VAX-ID groups. VAX-ID: product of vaccine-attitude and identity-centrality scores; T1: time 1; T2: time 2. Error bars show 95% confidence intervals.

Vaccine hesitancy data are shown in Fig 6. The ANOVA yielded the expected main effect of VAX-ID group *F*(1, 245) = 126.92, *p* < .001, η_p_^2^ = .341, but no other main effects, both *F* < 1. There was a marginal time by condition interaction, *F*(4, 245) = 2.50, *p* = .043, η_p_^2^ = .039, but no other significant interactions, all *F* < 1. No contrasts were significant (see Table 2, bottom section).

#### Analyses considering parent status

Next, analysis focused on participants with children, to allow comparison with Pluviano et al. (2019), who found a familiarity backfire effect in a parent sample. To this end, full-factorial repeated measures ANCOVAs were conducted with the within-subjects factor time (T1, T2), the between-subjects factors condition (control, myths only, myths vs. facts, visual, fear appeal) and parent status (yes, no), and VAX-ID as a covariate. (Note, this analysis was deemed appropriate despite unequal sample sizes, as ANOVA is relatively robust to sample size differences as long as variances are not also unequal. Nevertheless, analyses were repeated with equal sample sizes using a random subsample of non-parents; results were comparable.) There were no significant main effects or interactions involving parent status across all three dependent variables, all *F*(1/4, 360) ≤ 2.15, *p* ≥ .074, η_p_^2^ ≤ .023; this indicates that the effect of the experimental manipulations did not differ as a function of parent status. There were no backfire effects in any condition at any timepoint (see S2 Table in S1 File).

#### Replication using only Pluviano et al.’s (2017) items

Next, we replicated the original Pluviano et al. (2017) analyses using their one-item measures; the myths-only condition and the VAX-ID covariate were dropped for these analyses as they were not part of the original design. Separate time (T1, T2) by condition (control, myths vs. facts, visual, fear appeal) ANOVAs on the three dependent measures yielded no significant main effects of condition, all *F*(3, 297) ≤ 1.38, *p* ≥ .248, η_p_^2^ ≤ .014, and no time by condition interactions, all *F*(3, 297) ≤ 1.43, *p* ≥ .234, η_p_^2^ ≤ .014. There were no backfire effects in any condition at any timepoint (see S3 Table in S1 File).

#### Pre-post analyses

**Fig 7:**
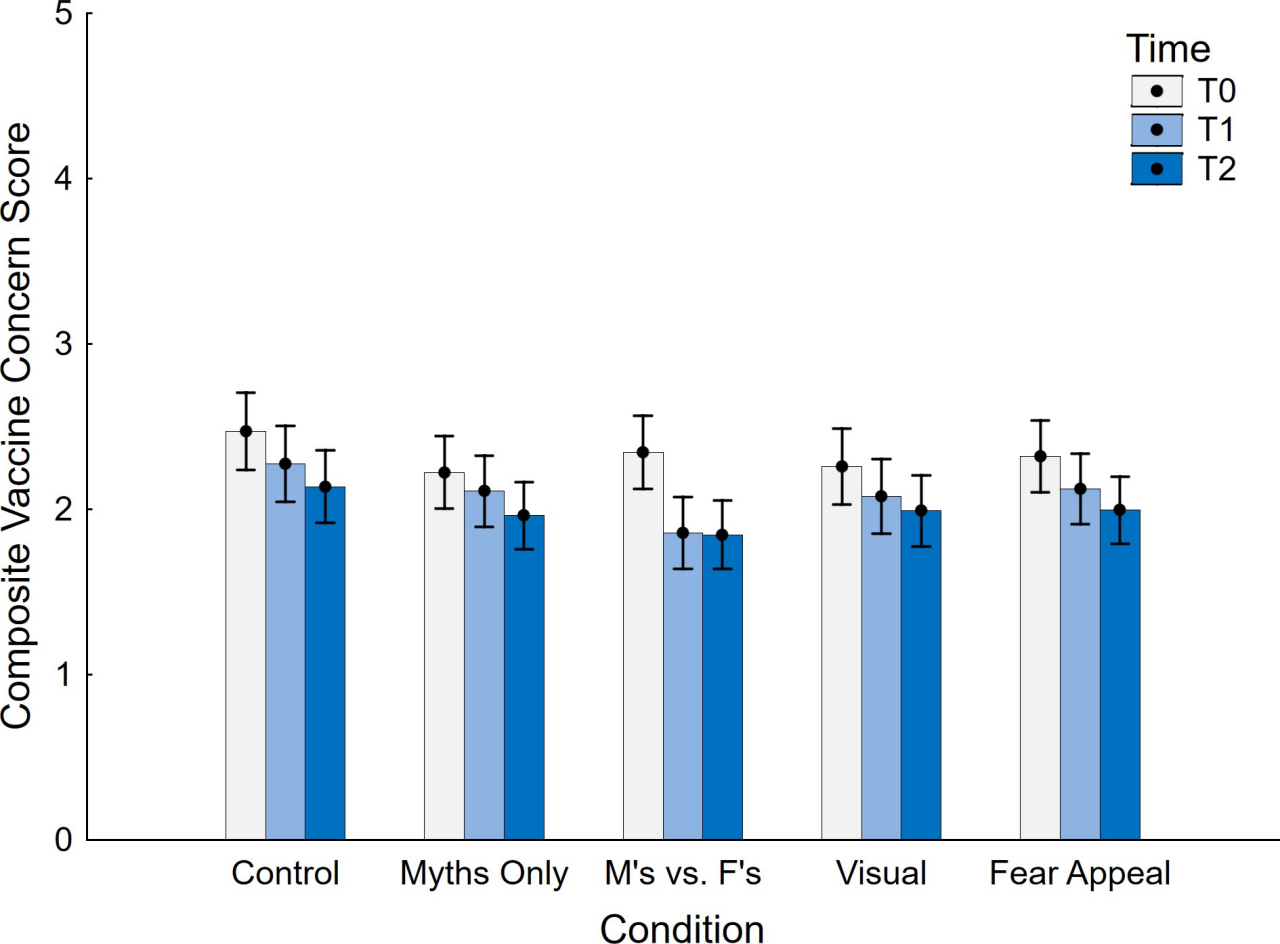
Pre- and post-intervention vaccine concern across conditions. M’s vs. F’s: Myths vs. Facts; T0: time 0 (pre-intervention); T1: time 1; T2: time 2. Error bars show 95% confidence intervals.

Backfire effects are defined by an ironic increase in belief relative to either a control condition (as used in the primary analyses) or a pre-manipulation baseline (Swire-Thompson et al., 2020). We therefore conducted pre-post analyses, using a composite measure based on the three items administered at all time points (T0, T1, T2). Data are shown in Fig 7. First, a between-subjects ANOVA with the sole factor of condition (control, myths only, myths vs. facts, visual, fear appeal) was conducted at T0, to ascertain that there were no condition differences at baseline; no differences between conditions were found, *F* < 1. Then, a full-factorial repeated-measures ANCOVA with the within-subject factor time (T0, T1, T2), the between-subjects factor condition (control, myths only, myths vs. facts, visual, fear appeal), and the VAX-ID covariate was conducted. This yielded a significant main effect of time *F*(2, 740) = 87.20, *p* < .001, η_p_^2^ = .191, indicating a significant decrease over timepoints. There was the expected main effect of VAX-ID, *F*(1, 370) = 518.51, *p* < .001, η_p_^2^ = .584, but no main effect of condition, *F* < 1. However, a significant interaction between time and condition was found, *F*(8, 740) = 3.35, *p* = .001, η_p_^2^ = .035, suggesting that the effect of the experimental manipulations varied across timepoints. There was also a time by VAX-ID interaction, *F*(2, 740) = 10.28, *p* < .001, η_p_^2^ = .027; closer inspection suggested this was due to stronger concern reduction over time in participants with greater VAX-ID scores (i.e., in those with greater vaccine concerns). There were no other significant effects, all *F* < 1.

Contrasts of T1 and T2, respectively, against T0 are presented in Table 3, separately for each condition. Aside from the myths-only condition at T1, all T1 and T2 scores were significantly lower than the T0 baseline, including the control condition. In line with the main analyses, when contrasted against control, only the myths-vs.-facts condition was associated with lower concern at T1, *F*(1, 375) = 12.61, *p* < .001, η_p_^2^ = .033, but not T2, *F*(1, 375) = 3.10, *p* = .079, η_p_^2^ = .008.

**Table 3:** Pre-post contrast analyses.

Contrast (vs. T0)	*F*(1, 375)	η_p_^2^	*p*
Control			
T1	10.70	.028	.001*
T2	23.52	.059	< .001*
Myths Only			
T1	4.11	.011	.043
T2	16.35	.042	< .001*
Myths vs. Facts			
T1	74.24	.165	< .001*
T2	58.97	.136	< .001*
Visual			
T1	9.41	.024	.002*
T2	15.70	.040	< .001*
Fear			
T1	12.67	.033	< .001*
T2	25.89	.065	< .001*

* indicates significance after Holm-Bonferroni correction

We finally examined the proportion of participants with numerically decreased misperceptions (corrective change), increased misperceptions (backfire), or no change post intervention. Proportions across conditions are summarized in Table 4.

**Table 4:** Proportions of numerical change tendencies (in %) across conditions and timepoints.

Numerical Change (from T0)	Corrective	No Change	Backfire
Control			
T1	54.29	25.71	20.00
T2	65.71	24.29	10.00
Myths Only			
T1	43.04	36.71	20.25
T2	55.70	27.85	16.46
Myths vs. Facts			
T1	65.38	29.49	5.13
T2	66.67	21.79	11.54
Visual			
T1	52.78	27.78	19.44
T2	58.33	23.61	18.06
Fear			
T1	55.56	29.63	14.81
T2	62.96	22.22	14.81

## Discussion

Despite a growing number of studies finding evidence against familiarity backfire effects [13, 26–29, 32, 33], several studies still claim familiarity to be a genuine mechanism for backfire effects [e.g., 22, 31]; given the sound theoretical reasons to believe familiarity backfire effects can occur, more solid evidence is required. Moreover, the concept still creates concern amongst practitioners, and it is therefore important to scrutinize reports of the effect. The present study therefore replicated and extended a study by Pluviano et al. (2017), which found that fear appeals and corrections presented in a myths-vs.-facts format backfired, inadvertently increasing belief in vaccine misconceptions and vaccination hesitancy relative to a control condition [10].

Contrary to Pluviano et al.’s [10] findings, based on the overall literature, it was predicted that corrections would reduce—not strengthen—false beliefs in vaccine side effects and the MMR-vaccine-autism link, as well as vaccination hesitancy, relative to control. We also expected corrections to reduce misconceptions and hesitancy (at timepoint T1) relative to a pre-intervention baseline (timepoint T0), although we expected some potential belief regression over time (at timepoint T2 relative to T1). However, this regression was not expected to reach or exceed baseline levels pre-intervention (at T0).

Results largely confirmed these predictions. We found that no intervention was associated with greater misinformation belief or vaccine hesitancy than control at any timepoint. This was true across all analyses and subgroups, including in parents (at odds with [34]) and in those participants higher in anti-vaccination sentiment (broadly in line with [54]). In the following, we focus our discussion on the impact of the interventions on misconceptions, before we briefly address their impact on vaccine hesitancy.

Although no condition *increased* vaccine misconceptions, only the myths-vs.-facts condition successfully *decreased* belief in vaccine side effects and the vaccine-autism link relative to control. When comparing pre- and post-intervention misconceptions, all conditions but the myths-only condition were associated with reduced misconceptions post-intervention, including the control condition. Again, it was only the myths-vs.-facts condition that led to a significantly stronger reduction than control, without belief regression back to baseline after a week. We acknowledge that the study had limited power to detect small effects (e.g., some observed non-significant effects were in the range of .01 < η_p_^2^ < .02), so some interventions may have been found to be significantly effective with greater power. This should not, however, distract from our core finding that no intervention demonstrated any tendency of backfire.

One reason for the efficacy of the myths-vs.-facts condition in decreasing misconceptions may lie in the clear and detailed alternative information presented when refuting the myths. It has been suggested that provision of alternative, factual information is the most important ingredient of a successful correction, allowing individuals to update their understanding and replace false with factual information in their mental models of the world [6, 8, 9, 35]. This also explains why no significant effect was found for the myths-only condition, which provided the weakest possible retraction [6, 12]. Despite the myths-only condition theoretically being the one most likely to cause a familiarity-driven backfire effect, though, no such effect was observed; this is particularly strong evidence against the notion of familiarity backfire.

In regard to the visual correction, its lack of efficacy relative to control was unexpected. It can be speculated that participants may not have actively engaged with the infographics to the extent required in order to allow a proper risk evaluation. While graphically-provided information has been shown to be effective, and potentially superior to text alone [36–40], extracting meaning from graphical material still requires individuals’ attention and engagement, even for low-level visual statistical learning [62]. The infographic used in the current study certainly did require attention to fully comprehend the colour coding and the relative-risk information conveyed. Infographics may thus only be useful for correcting misconceptions in situations where individuals are fully engaged with processing the information provided, or when the infographics are extremely simple. However, we again acknowledge that the study had limited power to detect small effects.

Finally, we did not find any evidence for a backfire effect in the fear-appeal condition either, which at the group level was also found ineffective relative to control. We note that this was not due to a pronounced bimodality (i.e., the intervention “working” for some participants but backfiring for others), as the backfire rate of approximately 15% was comparable to other conditions. The fact that intervention efficacy was relatively low overall, relative to control, is most likely associated with demand characteristics affecting responses in the control group. We note, however, that having a low level of misconception belief in the control condition will increase the likelihood of observing backfire effects. This is because the low belief in the control condition would leave sufficient leeway on the scale for the level of belief to surpass control in the other conditions.

Overall, the observed impact of the myths-vs.-facts condition is in line with recent evidence that has likewise found the format to be particularly efficacious, especially when compared to interventions that focus only on the facts without directly countering the myths [63, 64]. However, alternative formats should not be neglected based on current findings. As Swire-Thompson et al. [63] discuss, the optimal format may depend on the specific content and context of the correction, and in general it is more important *that* a myth is corrected than what specific format is used (also see [65]). More work is required to ascertain the relative strengths of different formats, including visual corrections that have been shown to be effective in other contexts.

With regards to the interventions’ impact on vaccine hesitancy, it was found that no intervention reduced vaccine hesitancy relative to control, even in participants with relatively high baseline levels of hesitancy. This is important because arguably the ultimate goal of any debunking intervention is to reduce misconceptions in order to change behavioural choices and outcomes. It is well-known that changes to beliefs and attitudes tend to not translate to equivalent changes in behavioural intentions and behaviours [66, 67]. In fact, other research has found that misinformation corrections tend to have stronger impact on the targeted misconceptions than on related behaviours or behavioural intentions, including vaccination intentions [e.g., 2, 45, 68, 69]. In the present study, it is possible that the observed effects are true small effects that would have been statistically significant with greater power and potentially meaningful at scale. However, effect sizes were consistently smaller than η_p_^2^ = .01, and as such it is also possible that more than a brief one-off intervention is necessary to achieve any practically significant change in intentions and behaviours.

A remaining question is: Why did our findings differ from those of Pluviano et al. (2017) [10]? We offer several reasons. First, Pluviano et al. conducted their study in 2016, when skepticism towards childhood vaccines may have been somewhat greater than in 2021 [55]. The Pluviano et al. study also included participants from both the UK and Italy (whereas our participants were only from the UK), and it is possible that the Italian participants were particularly vaccine-skeptical [56]. The backfire effect observed by Pluviano et al. may have thus been driven by worldview rather than familiarity. This is perhaps even more likely given that Italy introduced mandatory childhood vaccinations in mid-2017 because of relatively low vaccination rates compared to other European countries [70]. This may not only highlight relatively greater vaccine skepticism in Italy (pre-2017), but also suggests that public discourse around the mandate may have polarized the Italian sample in Pluviano’s study (we thank one of the reviewers, Dr Aimee Challenger, for pointing out this policy change in Italy). However, it is important to note that we did not observe any backfire effects even in the more vaccine-skeptical participants. Furthermore, the multi-item measures implemented in the present study to assess misinformation reliance likely provided a more reliable measure than the single-item measures utilized by Pluviano et al. It has been suggested that this lack of reliability may be the primary mechanism driving observed backfire effects, and in fact, to the best of our knowledge, *all* backfire effects reported with vaccine-related stimuli have been elicited using single-item measures [23, 33]. Thus, Pluviano et al.’s finding may have simply been a false-positive, given that their sample size was significantly lower than the sample size in the present study (for a similar case, see [32]).

A clear applied implication from this research is therefore that the hesitancy surrounding repetition of misinformation during correction is largely unwarranted. In light of the broader literature, the repetition of misinformation within a correction may actually be beneficial rather than harmful. Repetition may increase the salience of the correction while also facilitating processes for conflict resolution and knowledge revision [13, 27, 63, 71]. Although contemporary guidelines for debunking myths have already recognized this [8, 9], the current study provides further evidence of the efficacy of corrections that repeat the to-be-corrected misinformation. Misinformation correction should therefore not be avoided because of fear of backfire effects, especially when it comes to important topics such as vaccinations—in the current pandemic, there is clear opportunity for our findings to be applied to misinformation regarding COVID-19 vaccinations. However, unnecessary repetition of misinformation should still be avoided as there is a risk that it will enhance familiarity without any added benefit [8, 31, 32]. Moreover, there will be situations in which misinformation should not be corrected at all, to avoid amplifying a disinformant and adopting their framing of an issue, or where the misinformation has little traction and thus presents low risk of harm [8, 9, 32, 72].

Some limitations of the present research should be acknowledged. Our sample was an online sample that was relatively low in skepticism towards childhood vaccines. This was not a major concern for the present research because its main focus was on familiarity effects, which should occur independent of vaccine attitudes. However, future studies might consider using a sample from a more vaccine-skeptical population, as it is known that those with strongly-held beliefs can be motivated to defend them, potentially weakening the effectiveness of misinformation corrections in some circumstances (but see [49] for evidence to the contrary and further discussion). From an applied perspective, focusing on vaccine-skeptical individuals would be useful because it is this very population that needs to be engaged if they are to be motivated to vaccinate. Another limitation is that only vaccination intention was measured, rather than actual uptake behaviour. As mentioned earlier, intentions do not consistently translate into action, and there are a range of factors beyond intention that determine and contribute to behaviour execution [66, 67]. It is therefore recommended that future research investigate the uptake of healthcare behaviours following misinformation correction. Finally, we acknowledge some misalignment between intervention materials in some conditions and the measures obtained; for example, the fear appeal did not specifically relate to belief in the vaccine-autism link. As this was a direct replication, this was largely outside of our control. However, such misalignment might represent a threat to internal validity. For example, we might reasonably assume that all conditions were affected equally be the demand characteristics of this study; however, this may not actually be the case given the abovementioned misalignment in some conditions. Future research should therefore re-assess the interventions and their relative efficacy in more targeted studies.

## Conclusion

This study sought to replicate the familiarity and fear-related backfire effects reported by Pluviano et al. (2017) [10]. We found no evidence to support the notion that misinformation repetition or fear appeals cause backfire effects. This suggests that the findings reported by Pluviano et al. were either worldview-driven or an artefact. This highlights the importance of reproducibility in psychological science [73]. The only intervention successful in reducing vaccine misconceptions was a myths-vs.-facts approach that repeated the to-be-corrected misinformation and juxtaposed it with alternative factual information. It is thus recommended that this approach is used to proactively counter vaccination misinformation where it is encountered.

## Supporting information

S1 File (DOCX)
