# Supplementary material for: Correcting vaccine misinformation: A failure to replicate familiarity or fear-driven backfire effects
Source: PLoS One. 2023 Apr 12;18(4):e0281140. doi: 10.1371/journal.pone.0281140 (PMC10096191; doi:10.1371/journal.pone.0281140)
Supplement: S1 File — (DOCX) [file pone.0281140.s001.docx]

All supplementary information can be found at <https://osf.io/dwyma/>
